# Study on the Fatigue Strength of Welding Line in Injection Molding Products under Different Tensile Conditions

**DOI:** 10.3390/mi13111890

**Published:** 2022-11-02

**Authors:** Pham Son Minh, Van-Thuc Nguyen, Vinh Tien Nguyen, Tran Minh The Uyen, Thanh Trung Do, Van Thanh Tien Nguyen

**Affiliations:** 1 HCMC University of Technology and Education, Ho Chi Minh City 71307, Vietnam; 2 Department of Industrial Engineering and Management, National Kaohsiung University of Science and Technology, Kaohsiung 80778, Taiwan; 3 Faculty of Mechanical Engineering, Industrial University of Ho Chi Minh City, Nguyen Van Bao Street, Ward 4, Go Vap District, Ho Chi Minh City 70000, Vietnam

**Keywords:** fatigue cycle, deformation, amplitude, frequency, injection molding

## Abstract

The fatigue performance of polypropylene (PP) at various amplitudes and frequencies on fatigue cycles under tensile test conditions is investigated in this study. The results show that increasing the frequency leads to a decrease in fatigue cycles due to increased cycle time. The decline rate can be divided into two stages, between 1 and 5 Hz. The first stage rapidly decreases fatigue performance as the frequency increases from 1 Hz to 2 Hz or 3 Hz. The second stage has a lower reduction rate, which occurs between 2 Hz or 3 Hz and 5 Hz due to the strengthening effect of increasing frequency. Furthermore, increasing the amplitude from 0.1 mm to 0.4 mm reduces the fatigue cycle due to the higher deformation rate. In summary, expanding both amplitude and frequency reduces the fatigue performance of the PP material. Moreover, according to the scanning electron microscope microstructure, increasing the frequency results in more microcracks in the polymer matrix.

## 1. Introduction

Due to the advantages of a short cycle time, high efficiency, and ease of automation, injection molding has become the dominant process for producing plastic products. The injection mold design’s quality directly impacts the actual mold structure and the final plastic products. The efficiency and quality of mold design have increased over the last few decades due to the widespread use of Computer Aided Design/Engineering (CAD/CAE) technology. Researchers in the field of injection mold design have recently focused on feed and cooling system design [1,2], optimal parting direction determination [3], parting line and parting surface generation [4,5], and core and cavity generation [6]. However, the automated generation of venting systems is barely covered in the literature.

**Figure 1:**
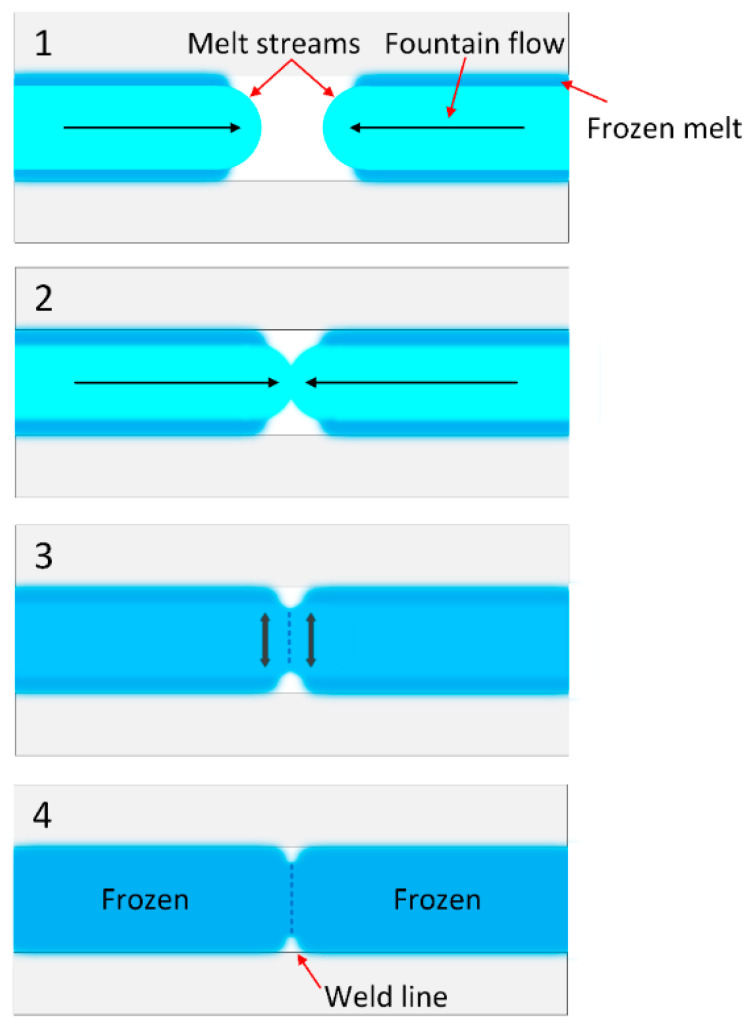
The weld line appearance in the injection molded part.

The weld line in injection molding is created when two melt streams connect, as shown in Figure 1. The melt stream is separated at the part’s cutout and reunited at the other end of the cutout. A weld line region is typically filled at the end of an injection stroke or during the pressure phase. The weld line’s strength is reduced when partially frozen melt fronts meet. The joint orientation remains perpendicular to the flow direction and signals weakening. Melting streams flowing in the same direction or opposite directions can form a weld line. Weld lines cannot be removed entirely, but they can be strengthened, or their position managed to change [7]. Numerous solutions have been proposed [8,9,10,11,12] for decreasing weld line problems and increasing weld line strength.

Polypropylene (PP) is a prevalent polymer material due to its advantages in mechanical, chemical, and physical properties [13,14]. PP is a lightweight thermoplastic polymer. It also has a high level of chemical resistance. Furthermore, PP can be manufactured using various techniques such as injection molding, extrusion, blowing, and compression [15,16]. PP is less expensive and more flexible for thermal forming than other common polymers such as polyethylene (PE), polystyrene (PS), polyethylene terephthalate (PET), and polyvinyl chloride (PVC) [17,18,19,20]. Other uses include fan blades, hand tools, bicycle wheels, and machine parts. These parts could be subjected to cyclic loads, sometimes in the form of low-amplitude vibrations over long periods, and consequently be vulnerable to fatigue failure in use. This issue raises questions about their functionality and long-term durability. Therefore, more significant consideration must be given to their design against fatigue failure. Some research has mentioned the fatigue strength of the weld line in the injection molding part when it stands by the stress [21,22,23,24,25,26,27,28,29,30]. However, in real applications, many plastic products will work under deformation, especially with the parts of microelectromechanical systems (MEMS). In these cases, the loading is not large; however, the deforming will let the material have creep deformation, and the fatigue stage will appear, which will decrease the plasticity of the part, as well as the weld line position.

Polymer materials may suffer severe damage due to the fatigue phenomenon after being used for an expected period under a moderate load [31,32,33]. The fatigue properties of composite materials containing a PP matrix and some reinforced fibers are widely studied [34,35,36]. For example, Ferreira et al. [37] investigated the fatigue properties of PP composites reinforced with glass fiber. This report demonstrated the superiority of the 0°-angle laminate over 30° and 45° angles. Gamstedt et al. [38] added maleic anhydride modification and discovered that it could strengthen the interfacial zone. As a result, it may improve the fatigue strength of the fiberglass-reinforced PP composite.

Interestingly, Petrucci et al. [39] revealed that adding 16% cotton and polyvinyl acetate binding agent could improve the fatigue strength of the PP composite. Mixing PP/wood flour composite with maleic anhydride and peroxide could enhance the tensile strength of this material [40]. However, the fatigue performance of the composite suffered a decline. In addition to fatigue, PP polymer could be degraded by the effects of sunlight and hydrolytic effects of the natural environment [41,42]. Moreover, PP and other common polymers are also impacted by heat, marine, and chemical conditions [43,44,45].

In addition, Bureau et al. [46] studied the impact of temperature on the fatigue performance of the fiberglass-reinforced PP composite. They reported that the fatigue value at 50 °C is lower than those at −40 °C and 23 °C due to the softening of the PP matrix. Reinforcing the PP matrix with alkali alone and alkali-silane led to a great improvement in the fatigue and impact characteristics due to the strengthening of the interfacial bonding [47]. Mixing additives, mainly fillers, results in higher costs and, in most cases, less flexibility in molding processes. The fatigue properties of PP under tensile conditions are rarely discussed and analyzed. Investigating the degradation of PP polymer is critical to predicting the product’s lifetime. Therefore, the outcome could be replaced in time, avoiding the severe damage that could lead to unsafe conditions.

Unlike previous studies, in this report, fatigue testing is applied to the welding line of the injection molding part. Moreover, the test occurs under different displacement amplitudes and frequencies, simulating the polymer products’ different loading conditions. The fatigue test is performed under repeated tensile requirements. The test sample is produced via an injection molding process with the composite material as PP mixed with CaCO_3_. According to Maiti et al. [48], adding CaCO_3_ powder to the PP matrix increases the elastic modulus. However, the tensile strength, impact toughness, and ductility values declined due to the debonding between the filler and the matrix and the weaker filler–matrix interface [49].

## 2. Experimental Methods

**Figure 2:**
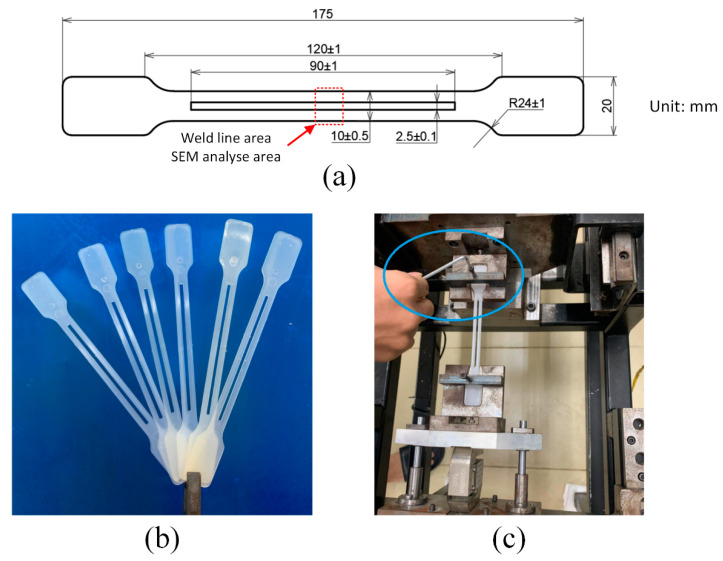
Testing samples under the standard of ASTM D638 (**a**), molded parts for testing (**b**), and fatigue testing machine (**c**).

The injection PP polymer was previously mixed with CaCO_3_ with five wt.% for improving the mechanical properties. The molding conditions are presented in Table 1. After injection molding with an ASTM D638 shape, the sample was assembled in a fatigue test machine, as shown in Figure 2. The fatigue test machine was controlled under Labview and DOPSoft software to manipulate the loadcell and the motor, respectively. The fatigue testing process will stop when the plasticity force drops 10% compared to the initial value. The sample surface was analyzed by scanning electron microscope JEOL 5410 LV (JEOL Ltd., Japan Office, Otemachi Nomura Bldg.13F 2-1-1, Otemachi, Chiyoda, Tokyo 100-0004, Japan). The observing points were the weld line in the middle of the sample, as shown in Figure 2a.

**Figure 3:**
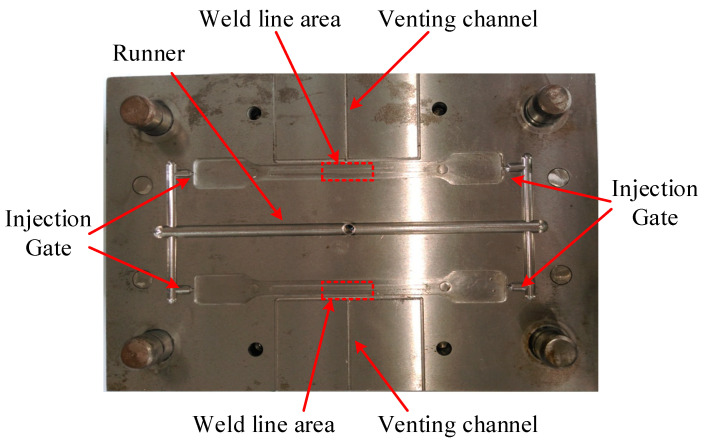
The cavity plate for molding the testing part.

**Figure 4:**
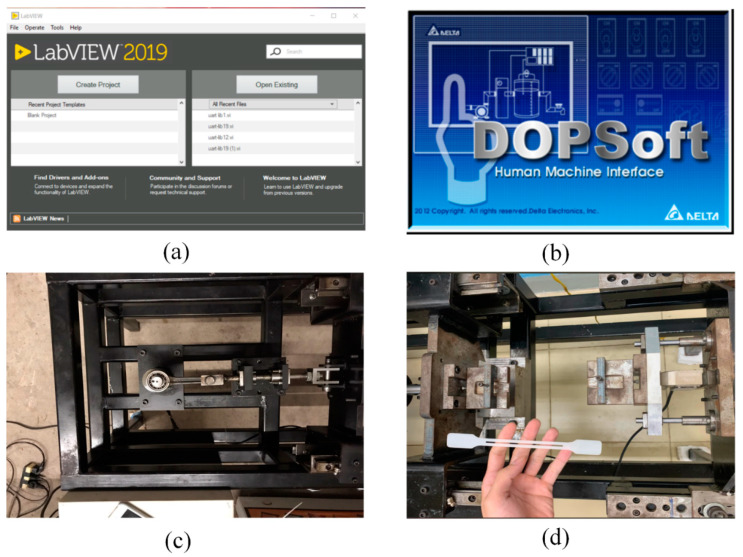
The experimental setup of the test: the LabVIEW software for controlling the loadcell (**a**), the DOPSoft software version 1.00.07, Delta Electronics Taiwan for managing the motor (**b**), the fatigue machine without a sample (**c**), and the fatigue sample before assembly (**d**).

For molding the testing sample, the mold structure was manufactured as shown in Figure 3 with the part size in Figure 2a. In this design, two cavities were created for the molding cycle. In each cavity, two gates were designed for generating the welding line at the “weld line area” as in Figure 3. The experimental setup is displayed in Figure 4. To reduce the negative influence of the air trap, the venting channel was designed to remove the air in the cavity volume.

## 3. Results and Discussion

### 3.1. Fatigue at Different Amplitudes

The PP samples are tested with different amplitudes of 0.1–0.4 mm, the fatigue frequency is set at 1–5 Hz, and each fatigue test condition is investigated via five samples.

**Figure 5:**
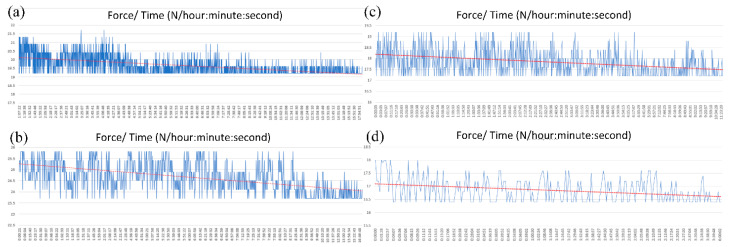
Fatigue diagrams of fatigue samples: sample 1.1 (**a**), sample 6.1 (**b**), sample 11.1 (**c**), and sample 16.1 (**d**).

Figure 5 shows the fatigue diagram of samples with different amplitudes and 1 Hz frequency. The force tends to reduce as the cycle time increases gradually. The amplitude of the force also suffers a decline when the cycle time increases. The reason for these reductions is the fatigue of the PP sample. The fatigue test will stop counting when the force drops 10% compared to the initial value during the testing process. The results show that sample 1.1, with an amplitude of 0.1 mm, is measured for about 25 h and 56 min, which is equal to 1556 fatigue cycles. Sample 6.1, with an amplitude of 0.2 mm, is estimated for approximately 17 h and 54 min, or 1073 fatigue cycles. Sample 11.1, with an amplitude of 0.3 mm, is measured for about 11 h 43 min, which equates to 703 fatigue cycles. Finally, sample 11.1, with an amplitude of 0.4 mm, has a fatigue performance of about 6 h 36 min, equal to 396 fatigue cycles. These fatigue cycle results reveal that increasing the amplitude causes a reduction in the fatigue cycles. Interestingly, the force diagram becomes sparser at a higher amplitude than those of lower amplitudes.

The test results of other PP samples with different amplitudes and 1 Hz frequencies are presented in Table 2. The average fatigue cycle value of the samples with 0.1 mm amplitude and 1 Hz frequency is 1488, 1058, 718, and 374, corresponding to the amplitudes of 0.1 mm, 0.2 mm, 0.3 mm, and 0.4 mm, respectively. The results indicate that increasing the amplitude leads to a decline in fatigue strength, because the higher amplitude generates a higher force and deformation rate, resulting in lower fatigue performance [50,51]. The samples suffer more severe damage during the fatigue test. In addition, we employ optimal design approaches by using machine learning to determine which parameters or factors affect fatigue [52,53,54,55,56] as additional tasks.

**Figure 6:**
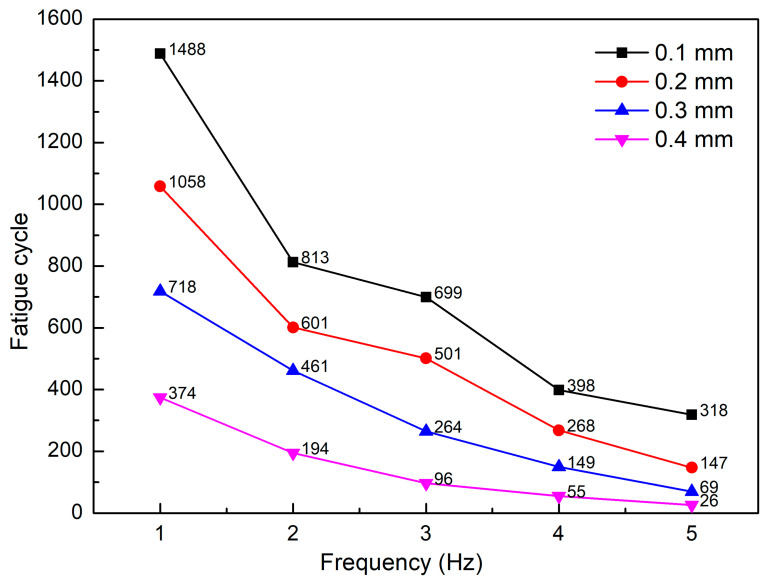
Fatigue cycle vs. frequency of PP samples at different amplitudes and frequencies.

Figure 6 represents the fatigue cycle diagram of PP samples tested at different amplitudes and frequencies. With 0.1 mm amplitude, from 1 Hz to 2 Hz, increasing the frequency leads to a sudden drop in the fatigue strength from 1488 cycles to 813 cycles. From 2 Hz to 5 Hz, the fatigue performance also decreases from 813 cycles to 318 cycles when increasing the frequency. However, the decreasing level is significantly lower than in the prior stage. The reason for this lower level could be the strengthening effect when increasing the frequency, a result consistent with that in Eftekhari et al.’s report [52]. In general, with the same frequency, increasing the amplitude from 0.1 mm to 0.4 mm decreases the fatigue performance due to the higher deformation rate. With the sample amplitude, increasing the frequency also leads to a decline in the fatigue performance of the PP sample. This reduction rate could be divided into two stages. The first stage indicates a rapid reduction in fatigue performance. This immediate reduction stage occurs when increasing the frequency from 1 Hz to 2 Hz or 3 Hz. The second stage has a lower reduction rate of fatigue per performance, which appears in the ranges from 2 Hz or 3 Hz to 5 Hz. In this range, the decreasing level is lower at the higher frequency.

### 3.2. SEM Microstructure

**Figure 7:**
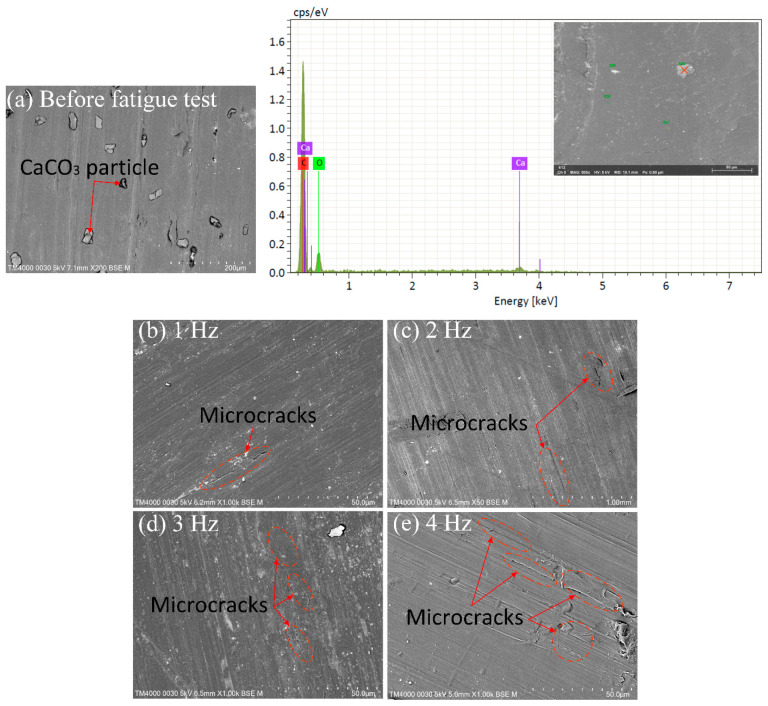
SEM microstructure and EDS spectra of PP samples before and after fatigue at 0.4 mm amplitude and different frequencies: (**a**) SEM microstructure before testing and EDS spectra, (**b**) 1 Hz, (**c**) 2 Hz, (**d**) 3 Hz, and (**e**) 4 Hz.

Figure 7 presents the SEM microstructure and the EDS spectra of PP samples before and after the fatigue test at 0.4 mm amplitude and different frequencies. These SEM figures display the middle point of the gauge length of the testing samples, which suffers the highest level of deformation during testing. Before the fatigue test, there are no microcracks on the sample surface, as shown in Figure 7a. Figure 7a also presents the results of the EDS spectra of particles dispersed in the PP matrix. The results show that these particles are CaCO_3_, as also mentioned in Section 2. CaCO_3_ particles are well-dispersed in the PP matrix. After the fatigue test, the microcracks appear in the sample, indicating the degradation of the materials. Increasing the fatigue frequency leads to more microcracks at the polymer matrix due to the higher deformation speed. This result is consistent with the fatigue cycle diagram in Figure 6. It indicates that the fatigue performance drops when the frequency increase as the samples suffer a higher deformation rate.

## 4. Conclusions

This study investigates the fatigue performance of PP under tensile test conditions. The analysis surveys the effects of different amplitudes and frequencies on the fatigue cycles. Some critical points are discussed as follows:

(i). Increasing the frequency leads to a decline in the fatigue cycles because of the increase in the cycle times. The maximum fatigue performance is 1488 cycles at 0.1 amplitude and 1 Hz frequency, while the minimum fatigue performance is 26 cycles at 0.4 amplitude and 5 Hz frequency.
(ii). The greater deformation rate, improving the amplitude from 0.1 mm to 0.4 mm, also decreases the fatigue cycles. Increasing both amplitude and frequency leads to a decline in fatigue performance.
(iii). The SEM microstructure shows that increasing the frequency leads to more microcracks on the polymer matrix. In the future, we plan to investigate the effects of injection conditions, such as a preheated mold, on the formation and strengthening of weld lines, to improve the sample’s fatigue strength.

## Figures and Tables

**Table 1:** Molding conditions.

Molding Parameters	Unit	Value
Melt temperature	°C	220
Mold temperature	°C	75
Injection pressure	MPa	40
Injection time	s	0.5
Drying time (85 °C)	h	12

**Table 2:** The fatigue cycle of PP samples with different amplitudes and 1 Hz frequency.

**Sample—0.1 mm**	**Time**	**Fatigue Cycle**	**Sample—0.3 mm**	**Time**	**Fatigue Cycle**
Sample 1.1	25:56:29	1556	Sample 11.1	11:43:18	703
Sample 1.2	24:01:32	1442	Sample 11.2	12:57:52	777
Sample 1.3	23:46:17	1426	Sample 11.3	11:09:58	669
Sample 1.4	25:54:20	1554	Sample 11.4	12:12:52	732
Sample 1.5	24:20:26	1460	Sample 11.5	11:51:35	711
Average		1488	Average		718
**Sample—0.2 mm**	**Time**	**Fatigue Cycle**	**Sample—0.4 mm**	**Time**	**Fatigue Cycle**
Sample 6.1	17:53:57	1073	Sample 16.1	6:36:07	396
Sample 6.2	17:27:02	1047	Sample 16.2	6:52:14	412
Sample 6.3	17:08:06	1028	Sample 16.3	6:07:05	367
Sample 6.4	17:46:05	1066	Sample 16.4	5:53:04	353
Sample 6.5	17:58:08	1078	Sample 16.5	5:40:30	340
Average		1058	Average		374

## Data Availability

The data used to support the findings of this study are available from the corresponding author upon request.
